# Cognitive Dysfunction Is Associated with Greater Imbalance and Falls in Essential Tremor

**DOI:** 10.3389/fneur.2017.00154

**Published:** 2017-04-19

**Authors:** Elan D. Louis, Sarah Kellner, Sarah Morgan, Kathleen Collins, Brittany Rohl, Edward D. Huey, Stephanie Cosentino

**Affiliations:** ^1^Division of Movement Disorders, Department of Neurology, Yale School of Medicine, Yale University, New Haven, CT, USA; ^2^Department of Chronic Disease Epidemiology, Yale School of Public Health, Yale University, New Haven, CT, USA; ^3^Center for Neuroepidemiology and Clinical Neurological Research, Yale School of Medicine, Yale University, New Haven, CT, USA; ^4^Department of Neurology, College of Physicians and Surgeons, Columbia University, New York, NY, USA; ^5^Taub Institute for Research on Alzheimer’s Disease and the Aging Brain, College of Physicians and Surgeons, Columbia University, New York, NY, USA; ^6^Division of Geriatric Psychiatry, Department of Psychiatry, College of Physicians and Surgeons and New York Psychiatric Institute, New York, NY, USA

**Keywords:** essential tremor, cognition, gait, balance, falls, clinical

## Abstract

**Background:**

Essential tremor (ET) is not exclusively a tremor disorder; it is also associated with cognitive and gait dysfunction. However, a gap in knowledge is that the relationship between cognitive and gait dysfunction has not been studied in detail in ET. We examined the relationship between cognition and balance and falls in ET and hypothesized that cognitive dysfunction in ET patients would be associated with greater problems with balance and more falls.

**Methods:**

ET cases were recruited into the Clinical–pathological Study of Cognition in ET. A comprehensive cognitive assessment was performed. This included the Montreal Cognitive Assessment (MoCA) to measure global cognition, multiple motor-free tests comprehensively assessing performance in each cognitive domain, and an assignment of Clinical Dementia Rating (CDR) scores. We collected data on the number of reported falls in the past year, and balance confidence was assessed using the 6-item Activities of Balance Confidence Scale. These cross-sectional analyses utilized baseline data.

**Results:**

There were 199 ET cases (mean age 78.6 years). In linear regression models that considered the effects of numerous confounding variables, lower global cognition (poorer cognition) was associated with greater number of falls and reduced balance confidence (*p* < 0.05). In similar adjusted linear regression models, higher CDR score (poorer functional cognition) was associated with greater number of falls and reduced balance confidence (*p* < 0.05). We also assessed whether number of falls and balance confidence was associated with performance in specific cognitive domains. Number of falls was most closely linked with performance on tests of executive function, and balance confidence, with executive function, attention, and memory.

**Conclusion:**

These data indicate that a correlate of poorer cognition in ET is greater number of falls and lower balance confidence. Cognition should enter the dialog with ET patients as an issue of clinical significance.

## Introduction

Essential tremor (ET) is not exclusively a motor disorder; it is associated in several respects with cognitive dysfunction. Numerous studies have reported the presence of mild cognitive deficits in ET cases compared with age-matched controls (1, 2). Cross-sectional epidemiological studies have further demonstrated associations between ET and both mild cognitive impairment (MCI) and dementia (3, 4). Additionally, prospective epidemiological studies have reported an increased risk of incident dementia among patients with ET (5, 6). Moreover, in a population-based study of 208 ET patients, lower cognitive test scores were associated with greater functional difficulty, indicating that such scores, rather than being inconsequential, have a clear clinical-functional correlate (7). Two additional clinical studies demonstrated that poorer cognitive performance was associated with lower activities of daily living scores (8, 9), further linking cognitive impairment with functional disability in ET.

Essential tremor patients also have gait and balance difficulties (10–12). This has been observed as a problem in tandem gait in some studies, as well as broader problems in postural control and balance in others (10–12). The problem is generally mild, although in some cases it can be severe and may have functional correlates (13).

Functional ambulation requires concurrent performance of motor and cognitive tasks. Studies of patients with Alzheimer’s disease (AD) (14) and Parkinson’s disease (PD) (15) have demonstrated that there are cognitive contributions to gait difficulties. Furthermore, poor judgment, which may occur in the setting of cognitive deficits, can result in patients putting themselves in situations which increase their risk for falling.

While cognitive and gait dysfunctions are both features of ET, at present, the relationship, if any, between cognitive and gait dysfunction has not been studied in detail in ET and hence, this represents a gap in knowledge. As detailed below, ET patients with a wide range of cognitive presentations, from normal cognition to dementia, were enrolled in a prospective, clinical–pathological study that utilized a comprehensive neuropsychological battery. In these analyses, which used baseline data, we cross-sectionally examined the relationship between cognitive performance and gait. We hypothesized that a correlate of cognitive dysfunction in patients with ET would be greater problems with balance and more falls. If true, this would further highlight the clinical significance of cognitive problems in ET.

## Materials and Methods

### Cases

Two hundred thirty-two ET cases were recruited into the Clinical–pathological Study of Cognition in Essential Tremor (NIH R01 NS086736). Baseline assessments have been completed (August 2014–November 2016), and prospective follow-up assessments are planned. The current analyses are cross-sectional, utilizing baseline data. Cases were diagnosed by their local neurologist as having ET, and diagnoses were confirmed after enrollment based on a detailed history and videotaped neurological examination reviewed by a senior neurologist specializing in movement disorders (EDL) who applied reliable and valid diagnostic criteria, as described below.

### Collection of Demographic and Clinical Data

Cases were evaluated in person by a trained tester who administered structured clinical questionnaires that elicited demographic and clinical information, including a detailed tremor history.

During the evaluation, all current medications were recorded, including medications that have the potential to affect cognitive performance (e.g., topiramate, primidone, and diazepam); for each case, the number of such medications was coded.

### Videotaped Neurological Examination and Confirmation of ET Diagnoses

As in our previous studies (16), each case underwent a videotaped neurological examination, which included a detailed assessment of postural tremor, five tests for kinetic tremor, and the motor portion of the Unified Parkinson’s Disease Rating Scale (17) excluding an assessment of rigidity. A senior movement disorders neurologist reviewed all videotaped examinations. Severity of postural and kinetic tremors were rated (0–3), resulting in a total tremor score [range 0–36 (maximum)], a measure of the severity of the action tremor. Based on the videotaped examination, the senior movement disorders neurologist confirmed ET diagnoses using published diagnostic criteria (moderate or greater amplitude kinetic tremor on ≥3 tests, or head tremor, in the absence of PD or dystonia) (18). None of the cases had co-occurring orthostatic tremor.

### Neuropsychological Evaluation

The neuropsychological evaluation included a comprehensive cognitive battery, a semi-structured interview with a close family member or friend informant, and clinical impressions of the case’s general cognitive function and functional abilities as reported by a trained tester. The cognitive battery included individual tests that were not dependent on efficient motor functioning, and which were grouped into the following cognitive domains: attention and working memory [Digit Span subtest from the Wechsler Adult Intelligence Scale-IV (WAIS) (19) and Symbol-Digit Modalities test (20)], executive function [Delis–Kaplan Executive System (21) subtests Verbal Fluency, Color-Word Interference, Sorting, and Twenty Questions], language [The Boston Naming Test (22) and the Multilingual Aphasia Examination Token subtest (23)], memory [California Verbal Learning Test—II (24), Verbal Paired Associates subtest from the Wechsler Memory Scale (WMS) (25) and the Logical Memory subtest A from the WMS-Revised Edition (26)], visuospatial abilities [Judgment of Line Orientation (27), Benton Facial Recognition (28), and the Visual Puzzles subtest of the WAIS-IV (26)], and global cognition [Mini-Mental State Examination (MMSE) (29) and Montreal Cognitive Assessment (MoCA) (30)].

### Gait Evaluation

We administered the 6-item Activities of Balance Confidence Scale (31, 32). The scale asks participants to rate their confidence in performing functional activities (e.g., reaching on tiptoes for an object, stepping on or off an escalator) without losing balance or becoming unsteady. Scores on each item range from 0 (not confident at all) to 100 (completely confident), and the total score ranges from 0 to 600. The 6-item version of the ABC scale has been validated against the parent ABC questionnaire, which consists of 16 items (31). In addition, we asked patients to indicate how many falls they had during the past year. Falls were defined as “an event which results in a person coming to rest inadvertently on the ground or supporting surface, and other than as a consequence of a violent blow, loss of consciousness or sudden onset of paralysis” (16).

### Cognitive Classification

Essential tremor cases were classified into cognitive groups (ET-normal cognition, ET-MCI, and ET dementia) through a consensus conference (neuropsychologist Stephanie Cosentino; neuropsychiatrist Edward D. Huey; and neurologist Elan D. Louis) using data from the semi-structured interviews with the patient and informant, and clinical and neuropsychological data. Clinical Dementia Rating (CDR) scores (33) were assigned based on the examiner’s interview and impression of the patient as well as informant report when available. Raw scores for cognitive tests were transformed to *z* scores based upon published normative data from healthy, cognitively intact controls, matched for age, education, and gender/ethnicity when possible. Impairment on neuropsychological tests was demonstrated by *z* scores of ≤−1.5 on at least two neuropsychological tests (either two tests in the same domain or one impaired test in two different domains).

Essential tremor-MCI diagnoses were based on criteria set forth by Petersen (34) and consisting of both impairment on testing as described above and functional deficit as reported by the participant or an informant (CDR of 0.5). Subjective memory complaint was not a requirement for the study or for the diagnoses. Cases with test impairment and CDR score ≥1 were considered demented. Cases with a CDR score <0.5 and no test impairment or impairment on only one test were considered ET-normal cognition.

### Final Case Selection

Two hundred thirty-two ET cases were recruited. However, for 24 ET cases, informants were not available for informant interviews, and hence, these 24 ET cases were excluded. Furthermore, MoCA scores or balance confidence scores were not available on 9. Hence, the final sample was 199 ET cases.

### Statistical Analyses

Statistical analyses were performed using SPSS (Version 24; Chicago, IL, USA). After reporting the demographic and clinical characteristics of cases (Table 1), we examined the clinical correlates of number of reported falls in the past year and balance confidence using Spearman’s correlation coefficients and Mann–Whitney tests (Table 2). Similarly, we examined the clinical correlates of the CDR and MoCA scores using Spearman’s correlation coefficients and Mann–Whitney tests (Table 3). In sensitivity analyses, we first restricted the sample to younger ET cases (i.e., <65 years of age, mean age = 59.2 ± 2.3 years, range = 55–63 years, *n* = 16) and ET cases with tremor of shorter (i.e., <10 years) duration (duration = 4.0 ± 2.5 years, *n* = 17). Since the sample size was very small in both samples, we assessed the direction (i.e., positive or negative) and magnitude of the correlation coefficient rather than the *p* value (Table 2).

**Table 1:** **Demographic and clinical characteristics of 199 essential tremor (ET) cases**.

Age (years)	78.6 ± 9.6 (range = 55–98)
Male gender	79 (39.7)
White race	195 (98.0)
Education (years)	15.8 ± 2.9
Age of tremor onset (years)	39.9 ± 22.8 (range = 1–89)
Tremor duration (years)	40.6 ± 22.3 (range = 3.7–88.4)
Total tremor score	24.2 ± 6.6
Number of reported falls in the past year	1.2 ± 2.5, median = 0.0 (range = 0–24)
Balance confidence (ABC-6)	332 ± 169, median = 352.5 (range = 0–594)
MoCA score	24.6 ± 3.9, median = 25.0 (range = 10–34)
CDR score	
None (0)	140 (70.4)
Questionable dementia (0.5)	46 (23.1)
Mild dementia (1)	9 (4.5)
Moderate dementia (2)	4 (2.0)
Number of medications that could affect cognitive performance	
0	97 (48.7)
1	62 (31.2)
2	28 (14.1)
3	7 (3.5)
4	5 (2.5)

*Values represent mean ± SD (range) or number (percentage)*.

*ABC-6, Activities of Balance Confidence Scale; CDR, Clinical Dementia Rating; MoCA, Montreal Cognitive Assessment*.

**Table 2:** **Clinical correlates of reported falls and balance confidence in 199 essential tremor (ET) cases**.

	Number of reported falls in the past year	Balance confidence
Age (years)	*r*_s_ = 0.11, *p* = 0.19	***r*_s_ = −0.34, ***p*** < 0.001**
Gender		
Men	1.3 ± 1.9 (1.0)	360.4 ± 166.7 (395.0)
Women	1.2 ± 2.9 (0.0)	313.1 ± 169.2 (310.0)
	*p* = 0.17^a^	*p* = 0.05^a^
Race		
White	1.3 ± 2.5 (0.0)	332.2 ± 169.6 (355.0)
Other	0.0 ± 0.0 (0.0)	321.8 ± 181.2 (245.0)
	NA	*p* = 0.88^a^
Education (years)	*r*_s_ = 0.01, *p* = 0.99	***r*_s_ = 0.15, ***p*** = 0.04**
Age of tremor onset (years)	*r*_s_ = 0.05, *p* = 0.47	*r*_s_ = −0.11, *p* = 0.12
Tremor duration (years)	*r*_s_ = 0.03, *p* = 0.73	*r*_s_ = −0.05, *p* = 0.51
Total tremor score	*r*_s_ = 0.07, *p* = 0.37	***r*_s_ = −0.18, ***p*** = 0.015**
Number of falls in the past year	–	***r*_s_ = −0.35, ***p*** < 0.001**
Balance confidence (ABC-6)	***r*_s_ = −0.35, ***p*** < 0.001**	–
MoCA score		
Entire sample (*n* = 199)	***r*_s_ = −0.19, ***p*** = 0.007**	***r*_s_ = 0.36, ***p*** < 0.001**
Younger cases (*n* = 16)	***r*_s_ = −0.43**	***r*_s_ = 0.39**
Cases of shorter duration (*n* = 17)	***r*_s_ = −0.38**	***r*_s_ = 0.45**
CDR score		
Entire sample (*n* = 199)	***r*_s_ = 0.23, ***p*** = 0.001**	***r*_s_ = −0.29, ***p*** < 0.001**
Younger cases (*n* = 16)	***r*_s_ = 0.58**	***r*_s_ = −0.37**
Cases of shorter duration (*n* = 17)	***r*_s_ = 0.34**	***r*_s_ = −0.43**
Number of medications that could affect cognitive performance	*r*_s_ = 0.08, *p* = 0.26	*r*_s_ = −0.08, *p* = 0.29

*Values are mean ± SD (median) or *r*_s_*.

*^a^Mann–Whitney test*.

*NA = statistical test not performed because all values in one group = 0*.

*Bolded values are significant*.

*ABC-6, Activities of Balance Confidence Scale; CDR, Clinical Dementia Rating; MoCA, Montreal Cognitive Assessment; r_s_, Spearman’s rho*.

**Table 3:** **Clinical correlates of CDR and MoCA scores in 199 essential tremor (ET) cases**.

	CDR score	MoCA score
Age (years)	***r*_s_ = 0.33, ***p*** < 0.001**	***r*_s_ = −0.46, ***p*** < 0.001**
Gender		
Men	0.3 ± 0.5 (0.0)	24.6 ± 4.0 (25.0)
Women	0.2 ± 0.3 (0.0)	24.6 ± 3.8 (25.0)
	*p* = 0.11^a^	*p* = 0.94^a^
Race		
White	0.2 ± 0.4 (0.0)	24.6 ± 3.9 (25.0)
Other	0.0 ± 0.0 (0.0)	26.0 ± 2.2 (25.5)
	*p* = 0.20^a^	*p* = 0.59^a^
Education (years)	*r*_s_ = −0.04, *p* = 0.54	*r*_s_ = 0.12, *p* = 0.10
Age of tremor onset (years)	*r*_s_ = 0.03, *p* = 0.67	*r*_s_ = −0.13, *p* = 0.07
Tremor duration (years)	*r*_s_ = 0.11, *p* = 0.13	*r*_s_ = −0.05, *p* = 0.49
Total tremor score	***r*_s_ = 0.21, ***p*** = 0.005**	***r*_s_ = −0.23, ***p*** = 0.002**
Number of reported falls in the past year	***r*_s_ = 0.23, ***p*** = 0.001**	***r*_s_ = −0.19, ***p*** = 0.007**
Balance confidence (ABC-6)	***r*_s_ = −0.29, ***p*** < 0.001**	***r*_s_ = 0.36, ***p*** < 0.001**
MoCA score	***r*_s_ = −0.62, ***p*** < 0.001**	–
CDR score	–	***r*_s_ = −0.62, ***p*** < 0.001**
Number of medications that could affect cognitive performance	***r*_s_ = 0.37, ***p*** < 0.001**	***r*_s_ = −0.30, ***p*** < 0.001**

*Values are mean ± SD (median) or *r*_s_*.

*^a^Mann–Whitney test*.

*Bolded values are significant*.

*ABC-6, Activities of Balance Confidence Scale; CDR, Clinical Dementia Rating; MoCA, Montreal Cognitive Assessment; r_s_, Spearman’s rho*.

In linear regression models, we examined the association between MoCA score and balance confidence. Because balance confidence was not normally distributed, we transformed the variable by taking the absolute value of 1—balance confidence (Table 4). In these models, assumptions of linearity, independence, homoscedasticity, and normality were met. In similar models, we examined the associations between CDR score and the transformed balance confidence variable (Table 4). In these models, assumptions of linearity, independence, homoscedasticity, and normality were also met. Because neither the number of reported falls nor its transformed variable was normally distributed, the dependent variable was fallers, defined as individuals with more than one fall in the past year (Table 4). In logistic regression models, we examined the association between MoCA score and fallers (Table 4). In similar models, we examined the associations between CDR score and fallers (Table 4). In all linear and logistic regression analyses, we first began with unadjusted models and then considered the effects of confounding variables. Model 1 used less restrictive criteria for confounding [an association (*p* < 0.05) between the confounding variables and either the independent or dependent variable]. Model 2 used more restrictive criteria for confounding [an association (*p* < 0.05) between the confounding variables and both the independent and dependent variables] (Table 4). Using Spearman’s correlation coefficients, we also assessed whether neuropsychological test scores within different domains (attention, executive function, language, memory, and visuospatial function) were associated with number of reported falls and balance confidence (Table 5).

**Table 4:** **Association between MoCA score, CDR score and number of reported falls and balance confidence in regression models**.

	Fallers (OR, 95% CI), *p* value	Balance confidence (ABC-6)
MoCA score		
Unadjusted model	**0.87 (0.80–0.95), ***p*** = 0.001**	**beta = 14.63, ***p*** < 0.001**
Adjusted model 1^e^	**0.86 (0.78–0.95), ***p*** = 0.004**^a^	**beta = 9.57, ***p*** = 0.005**^c^
Adjusted model 2^f^	**0.87 (0.80–0.95), ***p*** = 0.001**^b^	**beta = 10.15, ***p*** = 0.003**^d^
Unadjusted model^g^	0.92 (0.82–1.02), *p* = 0.10	**beta = 14.98, ***p*** < 0.001**
CDR score		
Unadjusted model	**OR = 3.96 (1.69–9.28), ***p*** = 0.002**	**beta = −138.97, ***p*** < 0.001**
Adjusted model 1^e^	**OR = 3.95 (1.50–10.45), ***p*** = 0.006**^a^	**beta = −112.38, ***p*** = 0.001**^c^
Adjusted model 2^f^	**OR = 3.96 (1.69–9.28), ***p*** = 0.002**^b^	**beta = −109.07, ***p*** = 0.001**^d^
Unadjusted model^g^	OR = 3.43 (0.78–15.06), *p* = 0.10	**beta = −194.01, ***p*** = 0.001**

*^a^Adjusted for age, total tremor score, and number of medications that could affect cognitive performance*.

*^b^No covariates included in the model*.

*^c^Adjusted for age, total tremor score, education, and number of medications that could affect cognitive performance*.

*^d^Adjusted for age and total tremor score*.

*^e^Less restrictive model: model 1 used less restrictive criteria for confounding [an association (*p* < 0.05) between the confounding variable and either the independent or dependent variable]*.

*^f^More restrictive model: model 2 used more restrictive criteria for confounding [an association (*p* < 0.05) between the confounding variable and both the independent and dependent variables]*.

*^g^Unadjusted model after having removed 13 cases with dementia*.

*Bolded values are significant*.

*ABC-6, Transformed Activities of Balance Confidence Scale score; CDR, Clinical Dementia Rating; CI, confidence interval; MoCA, Montreal Cognitive Assessment; OR, odds ratio*.

**Table 5:** **Association between specific cognitive variables and number of reported falls and balance confidence**.

	Number of reported falls in the past year	Balance confidence (ABC-6)
Attention		
Digit Span Forward—total correct (WAIS-IV)	*r*_s_ = −0.008, *p* = 0.91	***r*_s_ = 0.17, ***p*** = 0.015**
Digit Span Forward—length of longest (WAIS-IV)	*r*_s_ = −0.006, *p* = 0.93	***r*_s_ = 0.19, ***p*** = 0.009**
Symbol Digit Modality Test	***r*_s_ = −0.15, ***p*** = 0.03**	***r*_s_ = 0.18, ***p*** = 0.01**
Executive function		
Verbal Fluency (part D-KEFS)	***r*_s_ = −0.24, ***p*** = 0.001**	***r*_s_ = 0.24, ***p*** = 0.001**
Color-Word (part D-KEFS)	***r*_s_ = −0.28, ***p*** < 0.001**	***r*_s_ = 0.16, ***p*** = 0.03**
Sorting (part D-KEFS)	*r*_s_ = −0.09, *p* = 0.24	***r*_s_ = 0.30, ***p*** < 0.001**
Digit Span Backward—total correct (WAIS-IV)	***r*_s_ = −0.25, ***p*** < 0.001**	***r*_s_ = 0.18, ***p*** = 0.01**
Digit Span Backward—length of longest (WAIS-IV)	***r*_s_ = −0.22, ***p*** = 0.002**	***r*_s_ = 0.23, ***p*** = 0.001**
Twenty Questions (part D-KEFS)	*r*_s_ = −0.07, *p* = 0.34	***r*_s_ = 0.25, ***p*** = 0.001**
Language		
Boston Naming Test	*r*_s_ = −0.04, *p* = 0.54	***r*_s_ = 0.18, ***p*** = 0.01**
Token Test (MAE, third edition)	***r*_s_ = −0.16, ***p*** = 0.03**	*r*_s_ = 0.01, *p* = 0.90
Memory		
California Verbal Learning Test (Immediate)	***r*_s_ = −0.16, ***p*** = 0.03**	***r*_s_ = 0.31, ***p*** < 0.001**
California Verbal Learning Test (Long Delay)	***r*_s_ = −0.17, ***p*** = 0.02**	***r*_s_ = 0.32, ***p*** < 0.001**
Verbal Paired Associates (Immediate)	*r*_s_ = −0.10, *p* = 0.15	***r*_s_ = 0.18, ***p*** = 0.01**
Verbal Paired Associates (Delayed)	*r*_s_ = −0.09, *p* = 0.24	***r*_s_ = 0.22, ***p*** = 0.003**
Logical Memory (Immediate)	*r*_s_ = −0.12, *p* = 0.10	***r*_s_ = 0.15, ***p*** = 0.04**
Logical Memory (Delayed)	***r*_s_ = −0.16, ***p*** = 0.025**	***r*_s_ = 0.17, ***p*** = 0.016**
Visuospatial function		
Judgment of Line Orientation	*r*_s_ = 0.04, *p* = 0.71	*r*_s_ = 0.17, *p* = 0.08
Benton Facial Recognition Test	*r*_s_ = −0.11, *p* = 0.11	*r*_s_ = 0.01, *p* = 0.86
Visual Puzzles subtests of WAIS-IV	*r*_s_ = −0.10, *p* = 0.19	*r*_s_ = 0.11, *p* = 0.16

*Bolded values are significant*.

*ABC-6, Activities of Balance Confidence Scale; D-KEFS, Delis–Kaplan Executive System; MAE, Multilingual Aphasia Examination; r_s_, Spearman’s rho; WAIS, Wechsler Adult Intelligence Scale*.

## Results

### Case Characteristics

There were 199 ET cases; the mean age was 78.6 years but ranged from 55 to 98 years (Table 1). Age of tremor onset was ≥65 in 35 (17.6%), <65 in 159 (79.9%), and unknown in 5 (2.5%). The mean number of reported falls in the past year was 1.2 but ranged from 0 to 24. The mean balance confidence score was 332 but ranged from 0 to 594. Thirteen (6.5%) cases had ET dementia, and 32 (16.1%) had ET-MCI.

More reported falls in the past year was associated with lower balance confidence, a lower MoCA score (i.e., more cognitive impairment) and a higher CDR score (i.e., poorer functional cognition) (Table 2). Lower balance confidence was associated with older age, lower education, greater tremor severity, lower MoCA score (i.e., more cognitive impairment), and a higher CDR score (i.e., more cognitive impairment) (Table 2). In sensitivity analyses, we first restricted the sample to younger ET cases (i.e., <65 years of age, mean age = 59.2 ± 2.3 years, range = 55–63 years, *n* = 16) and ET cases with tremor of shorter (i.e., <10 years) duration (mean duration = 4.0 ± 2.5 years, *n* = 17). In both samples, more reported falls in the past year was associated with a lower MoCA score (i.e., more cognitive impairment) and a higher CDR score (i.e., poorer functional cognition), and lower balance confidence was associated with lower MoCA score (i.e., more cognitive impairment) and a higher CDR score (i.e., more cognitive impairment) (Table 2).

Higher CDR score (i.e., more cognitive impairment) was associated with older age, higher total tremor score, lower MoCA score (i.e., more cognitive impairment), and more medications that could affect cognitive performance (Table 3). Lower MoCA score (i.e., more cognitive impairment) was associated with older age, higher total tremor score, higher CDR score (i.e., more cognitive impairment), and more medications that could affect cognitive performance (Table 3).

In regression models that considered the effects of confounding variables, higher MoCA score (i.e., less cognitive impairment) was associated with lower odds of being a faller (Table 4) and increased balance confidence (Table 4). In similar models, higher CDR score (i.e., more cognitive impairment) was associated with greater odds of being a faller and reduced balance confidence (Table 4). When we removed 13 ET cases with dementia, the results were similar, although for fallers, the *p* values did not reach statistical significance (Table 4).

We also assessed whether a range of neuropsychological test scores within different domains (attention, executive function, language, memory, and visuospatial function) were associated with number of reported falls and balance confidence (Table 5). Number of reported falls in the past year was most closely linked with executive function and memory (Table 5). Balance confidence was linked with executive function, attention, and memory rather than visuospatial function (Table 5).

## Discussion

In this study of approximately 200 ET cases, we noted that lower cognitive test scores were associated with greater number of reported falls and reduced balance confidence. After removing the 13 ET cases with comorbid dementia, the results remained similar.

Prior work establishing links between cognitive performance and gait dysfunction in ET has been limited. In a study of 151 ET cases, the authors examined the extent of interference between gait and cognition in patients with ET during a dual-task gait test (35). Functional ambulation requires concurrent performance of motor and cognitive tasks, which may create interference (degraded performance) in either or both tasks (35). Therefore, the authors hypothesized that ET cases with lower cognitive scores would demonstrate greater cognitive-motor interference compared with ET cases with higher cognitive scores. Accordingly, ET cases were divided into two groups based on their median score on the modified MMSE; as hypothesized, ET cases with lower cognitive test scores demonstrated high levels of cognitive-motor interference (35). In another study, 132 ET cases were similarly divided based on their median modified MMSE scores (16). ET cases with lower scores had lower balance confidence and more reported falls than those with higher scores (16). That study differed from the current in the sense that the assessment was limited to a global screen for cognition rather than an extensive, motor-free neuropsychological test battery followed by a careful consensus diagnosis of MCI or dementia (16). Furthermore, ET cases were significantly older than those of the current cohort and their gait parameters therefore differed significantly (16).

We also assessed whether performance in separate cognitive domains (attention, executive function, language, memory, and visuospatial function) was associated with number of reported falls and balance confidence (Table 5). Number of reported falls in the past year was most closely linked with memory and executive function (Table 5). Balance confidence was linked with executive function, attention, and memory (Table 5). Previous work, in patients with PD and AD, similarly suggests that balance is most associated with executive dysfunction, although balance does rely on other cognitive domains, including visuospatial function (14, 36–38).

While cognitive function may influence functional gait, poor judgment, which may occur in the setting of cognitive deficits, can result in patients putting themselves in situations which increase their risk for falling. Falls may even occur when not walking as well (e.g., falling out of bed, falling while reaching for something). Another issue is that cognitively impaired patients are much less able than intact patients to use assistive devices such as a walker.

What are the clinical implications of this work? The data indicate that cognitive dysfunction in ET is not merely an incidental finding without clinical correlates. Greater cognitive dysfunction is associated with more reported falls and less confidence in balance. At the root of this could be the fact that functional ambulation requires concurrent use of motor and cognitive systems (35). The data offer additional proof of principle that cognition should enter the clinical dialog with ET patients, as it is an issue of clinical significance—ET patients with cognitive deficits could be at greater risk for falls with attendant injuries. Hence, there are additional reasons to assess the cognitive profile of ET patients. Other issues include the following: a patient with cognitive impairment may be unable or unwilling to use an assistive device or to participate in physical therapy or exercise training. Furthermore, falls may be an indication for more intensive monitoring of a cognitively impaired patient.

Our findings should be interpreted in the context of several limitations. First, our analyses were cross-sectional rather than longitudinal. Hence, we are unable to make inferences about temporal relations and potential causal relationships between cognitive deficits, imbalance and falls in ET. Furthermore, the relationship between the onset of cognitive and gait dysfunction and the ET diagnosis is not known. Our ongoing prospective follow-up assessments will eventually provide longitudinal data that will facilitate a more comprehensive set of analyses of these issues. Second, although our measures were commonly used measures of imbalance, they are likely susceptible to self-report bias in individuals with cognitive impairment. In an attempt to address this issue, we repeated our main analyses after having excluded all cases with dementia, and our results were unchanged. Self-report bias may be minimized in future work by also using objective measures of gait, such as quantitative gait analysis. Third, this study was not designed to explore the neuroimaging correlates of cognitive dysfunction, gait impairment, and their combination in ET. Such information would be of mechanistic interest in future studies.

This study also had several strengths. These included the enrollment and consensus diagnosis of ET cases with a full range of cognitive presentations from normal cognition through to dementia, our use of a comprehensive, motor-free neuropsychological examination rather than just a global cognitive screen, and our evaluation of multiple possible co-contributors to either cognition or imbalance.

Although tremor has been the defining element of ET, lower cognitive test scores are increasingly being recognized. However, on a scientific level, the clinical correlates, if any, of these lower test scores have been largely unexplored. The current data indicate that a correlate of poorer cognition in ET is a greater number other motor features, including falls and less balance confidence. Future studies that are longitudinal, that employ objective measures of gait, such as quantitative gait analysis, and that incorporate a neuroimaging component will facilitate greater scientific clarity. Cognition should enter the clinical dialog with ET patients as an issue of clinical significance.

## Ethics Statement

Upon enrollment, all cases provided informed written consent approved by the Institutional Review Boards of both Columbia University and Yale University.

## Author Contributions

EL: conception, organization, and execution of the research project; design and execution of the statistical analysis; and writing of the first draft of the manuscript; SK, SM, KC, and BR: organization and execution of the research project; review and critique of the manuscript; EH and SC: conception, organization, and execution of the research project; review and critique of the manuscript.

## Conflict of Interest Statement

The authors declare that the research was conducted in the absence of any commercial or financial relationships that could be construed as a potential conflict of interest.
